# COMP report: CPQR technical quality control guidelines for major dosimetry equipment

**DOI:** 10.1002/acm2.12346

**Published:** 2018-05-09

**Authors:** Gérard Lagmago Kamta

**Affiliations:** ^1^ CISSS Montérégie‐Centre Centre Intégré de Cancérologie de la Montérégie, Radiothérapie Greenfield Park QC Canada

**Keywords:** quality assurance, radiation dosimeters, radiation dosimetry equipments

## Abstract

The Canadian Organization of Medical Physicists (COMP), in close partnership with the Canadian Partnership for Quality Radiotherapy (CPQR) has developed a series of Technical Quality Control (TQC) guidelines for radiation treatment equipment. These guidelines outline the performance objectives that equipment should meet to ensure an acceptable level of radiation treatment quality. The TQC guidelines have been rigorously reviewed and field tested in a variety of Canadian radiation treatment facilities. The development process enables rapid review and update to keep the guidelines current with changes in technology (the most update version of this guideline can be found on the CPQR website). This article provides guidelines for quality control testing of major dosimetry equipment.

## 1 INTRODUCTION

The Canadian Partnership for Quality Radiotherapy (CPQR) is an alliance among the three key national professional organizations involved in the delivery of radiation treatment in Canada: the Canadian Association of Radiation Oncology (CARO), the Canadian Organization of Medical Physicists (COMP), and the Canadian Association of Medical Radiation Technologists (CAMRT). Financial and strategic backing is provided by the federal government through the Canadian Partnership Against Cancer (CPAC), a national resource for advancing cancer prevention and treatment. The mandate of the CPQR is to support the universal availability of high quality and safe radiotherapy for all Canadians through system performance improvement and the development of consensus‐based guidelines and indicators to aid in radiation treatment program development and evaluation.

This publication, *Technical Quality Control Guidelines for Major Dosimetry Equipment*, contains detailed performance objectives and safety criteria for Major Dosimetry Equipment. Please refer to the overarching document *Technical Quality Control Guidelines for Canadian Radiation Treatment Centres* 1 for a programmatic overview of technical quality control, and a description of how the performance objectives and criteria listed in this document should be interpreted. The development of the individual TQC guidelines is spearheaded by expert reviewers and involves broad stakeholder input from the medical physics and radiation oncology community.^2^

All information contained in this document is intended to be used at the discretion of each individual centre to help guide quality and safety program improvement. There are no legal standards supporting this document; specific federal or provincial regulations and licence conditions take precedence over the content of this document.

## 2 SYSTEM DESCRIPTION

### 2.A Ionization chambers and electrometers used for reference dosimetry

The absorbed dose to water at the reference point under reference conditions as specified in the appropriate dosimetry protocols^3^, ^4^, ^5^, ^6^ is determined through the use of a chamber/electrometer combination. Local or secondary standards are chamber/electrometer combinations which have a calibration coefficient in terms of absorbed dose directly traceable to a primary standards dosimetry laboratory (e.g., National Research Council of Canada [NRCC], National Institute of Standards and Technology [NIST], or an accredited dosimetry calibration laboratory). Redundancy for these devices is recommended to assure the maintenance of the calibration during, and following, calibration at the standards lab.^3^, ^4^, ^5^, ^6^ These standards, which comprise a unique chamber/electrometer combination, are the basis of accurate dose delivery and are generally removed from routine clinical use. Routine dose measurements and therapy device calibration in the clinical setting are typically performed with field grade chambers and electrometers (hereafter referred to as field standards), which have a calibration coefficient transferred from the secondary standard.

### 2.B Detectors for non‐reference dosimetry

These are detectors used to measure dose from a radiation source as a method of ensuring the stability of the device on a routine basis. They can also be used to determine the absolute dose in a phantom or received by a patient following a cross‐calibration process. Some of these devices in use include ionization chambers, diodes, thermoluminescent dosimeters (TLDs), metal‐oxide semiconductor field‐effect transistors (MOSFETs), optically stimulated luminescence (OSL) systems, scintillating fibre dosimeters, radiographic films,^7^ and radiochromic films.^8^ Both types of films are integral parts of routine quality assurance for intensity‐modulated radiation therapy (IMRT) treatment plans and for stereotactic radiosurgery.

### 2.C Basic measurement devices

Most secondary and field standards are vented ionization chambers and as such, are subject to local atmospheric conditions. Therefore, thermometers, barometers, and hygrometers will be used during reference dosimetry measurements. Basic distance checks will be achieved with a quality ruler or caliper. A quality stopwatch will be used for accurate time measurement. Spirit levels (with or without digital angle display) could be used for levelling scanning water tanks and other measurement phantoms or devices. A self‐adjusting laser system projecting two perpendicular laser lines may be used to check the horizontality and verticality of room lasers.

### 2.D Automated beam scanning devices

Automatic remotely controlled water scanners comprise a water tank and a mechanism for holding and moving a radiation detector through the beam. They range in sophistication from ion chamber motion/measurements along a single vertical axis (1D water tanks) to a motion along two (2D water tank) and three directions (3D water tanks). While 1D water tanks are mainly used for chamber positioning at a desired reference point for clinical reference dosimetry,^3^, ^4^, ^5^, ^6^ 3D water tanks are used for beam data acquisition in acceptance testing and commissioning of radiation therapy units, as well as for periodic checks of beam parameters such as flatness, symmetry, depth dose, off‐axis ratios and energy. These systems may also be capable of real‐time isodose tracking and dynamic beam measurement, and are equipped with software tools for plotting, analyzing, and applying various transformations (shifts, scale, move, smooth, etc.) on measured data, and for converting the ionization depth curves into dose according to various protocols.^3^, ^9^ Also available are smaller 3D scanning water tanks that fit into the gantry bore of tomotherapy units or that are adapted specifically for tissue‐phantom ratio (TPR) type measurements of stereotactic fields; these are subject to the same quality control tests as larger scanning water tanks.

### 2.E Machine quality assurance devices

Megavoltage beam parameters such as output, field size, flatness, symmetry, beam energy, and constancy can be measured on a routine basis with a variety of devices which are more convenient to use than the water scanner. These devices may consist of one or more two‐dimensional detector arrays of diodes or ionization chambers and may have software for processing, analyzing, and tracking measured data. These devices, which consist essentially of two‐dimensional detector arrays, are easy to setup and use, and their multi‐detector construction involving ion chamber and/or diodes makes them useful in the monitoring of technologies such as dynamic wedge and IMRT beam quality assurance.^10^, ^11^

### 2.F Treatment delivery quality assurance devices

Patients’ plans for static or rotational IMRT techniques often involve a pretreatment verification that the beam is delivered accurately and precisely with respect to the plan. In general, a phantom approach is used, whereby the treatment plan is transferred onto a phantom containing detectors, the dose is recalculated on the treatment planning system (TPS) for this phantom setup and the treatment plan is delivered on the phantom and measured for comparison with the TPS‐calculated dose. Various devices available for this pretreatment delivery quality assurance consist of 2D or 3D arrays of diodes or ionization chambers, and have additional hardware and software for instant readout, data manipulation, and analysis of measured doses versus the planned dose. In addition, some 2D arrays have features that can be used for machine quality assurance and also have accessories for mounting them on the linac gantry.

### 2.G Phantom materials

While water is the reference phantom material for clinical reference dosimetry, solid phantoms are typically used for routine measurement. These devices may have radiation absorption properties and interaction coefficients similar to water, and may also be available in other materials such as acrylic, bone, lung, or muscle. The phantom may have “slab” geometry or be anthropomorphic. Anthropomorphic or “humanoid” phantoms are often constructed so as to accommodate TLD, MOSFETs, and film measurements. Motion phantoms that incorporate various forms of detector or target movements are also available for assessing 4D imaging and treatment gating capabilities (see Tables 1, 2, 3, 4, 5, 6, 7).

**Table 1:** Reference dosimeters

Designator	Test	Performance	
		Tolerance	Action
Secondary standard (chamber and electrometer combination)			
Initial use and following calibration			
ISS1	Extracameral signal (stem effect)	0.5%	1.0%
ISS2	Ion collection efficiency	Characterize and document	
ISS3	Polarity correction	Characterize and document	
ISS4	Linearity	0.5%	1.0%
ISS5	Leakage	0.1%	0.2%
ISS6	Collection potential reproducibility	1.0%	2.0%
At each use			
ESS1	Signal reproducibility	0.2%	0.5%
Biennial			
BSS1	Calibration at standards lab	Every 2 yr	
Field standard (chamber and electrometer combination)			
Initial use or following malfunction and repair			
IFS1	Extracameral signal (stem effect)	0.5%	1.0%
IFS2	Ion collection efficiency	Characterize and document	
IFS3	Linearity	0.5%	1.0%
IFS4	Leakage	0.1%	0.2%
IFS5	Collection potential reproducibility	1.0%	2.0%
IFS6	Cross‐calibration	Characterize and document	
Annual			
AFS1	Signal reproducibility	0.2%	0.5%
AFS2	Collection potential reproducibility	1.0%	2.0%
AFS3	Cross‐calibration	Characterize and document	
Detector cables, connectors, and adaptors			
At each use			
ECC1	Integrity and functionality	Any defect	Any defect

Note:

ISS1–ISS6: Tolerances based on American Association of Physicists in Medicine (AAPM) TG‐40.^12^ Suggested methods for measurement of ion collection efficiency and polarity correction may be found in AAPM TG‐51.^3^ For flattening filter free (FFF) beams, the effects of higher dose rates should be investigated, as recommended by AAPM TG‐51 Addendum.^4^ Leakage tolerance and action levels are based on the ratio of leakage versus ionization current/charge. Since collection potential (voltage) is difficult to accurately measure with the chamber connected, the user may rely on the internal device readout for the measurement of the collection potential reproducibility test (ISS6).

ESS1, BSS1: Based on AAPM TG‐40.^12^

IFS1–IFS5: Tolerances based on AAPM TG‐40.^12^ Suggested methods for measurement may be found in AAPM TG‐51.^3^

IFS6: Based on clinical experience.

AFS1, AFS2: Based on clinical experience and AAPM TG‐40.^12^ Since collection potential (voltage) is difficult to accurately measure with the chamber connected, the user may rely on the internal device readout for the measurement of the collection potential reproducibility test.

AFS3: Modified frequency from AAPM TG‐40^12^ based on clinical experience.

ECC1: Prior to their use, detectors, cables, and connectors should be checked for any defects and for functionality. An unusually high leakage level or lack of reproducibility of measurements is an indication of a problem and would need to be addressed.

**Table 2:** Non‐reference dosimeters

Designator	Test	Performance	
		Tolerance	Action
Thermoluminescent dosimeter (TLD) systems			
Initial use or following malfunction and repair			
IRD1	Linearity or supralinearity	Characterize and document	
At each use			
ERD1	Individual absolute dose cross‐calibration	Characterize and document	
Radiographic and radiochromic film dosimetry systems			
Initial use or following malfunction and repair			
IRD2	Sensitometric curve	Characterize and document	
Weekly or longer depending on workload and usage			
WRD1	Film processor quality control	Manufacturer's recommendations	
Biennial or shorter depending on workload			
ARD1	Film reader linearity, reproducibility, and geometric accuracy	Characterize and document	
Ionization chambers for relative dosimetry			
Initial use or following malfunction and repair			
IRD3	Linearity (dose and dose rate)	0.5%	1.0%
IRD4	Extracameral signal (stem effect)	0.5%	1.0%
Annual			
ARD2	Signal reproducibility	0.5%	1.0%
Diode systems			
Initial use or following malfunction and repair			
IRD5	Linearity	Characterize and document	
IRD6	Energy dependence	Characterize and document	
Annual or shorter (depending on workload)			
ARD3	Absolute dose calibration (if required)	Characterize and document	
MOSFETs			
Initial use or following malfunction and repair			
IRD7	Energy dependence	Characterize and document	
IRD8	Absolute dose calibration	Characterize and document	
Annual or shorter (depending on workload)			
ARD4	Absolute dose calibration	Characterize and document	
Optically stimulated luminescence (OSL) systems			
Initial use or following malfunction and repair			
IRD9	Linearity	Characterize and document	
IRD10	Absolute dose calibration	Characterize and document	
Annual or shorter (depending on workload)			
ARD5	Absolute dose calibration	Characterize and document	
Scintillating fibre dosimeter (SFD) systems			
Initial use or following malfunction and repair			
IRD11	Linearity	Characterize and document	
IRD12	Absolute dose calibration	Characterize and document	
IRD13	Stem effect	0.5%	1.0%
Annual or shorter (depending on workload)			
ARD6	Absolute dose calibration	Characterize and document	

Note:

IRD1: Based on AAPM TG‐40.^12^ Investigation of linearity and supralinearity for a sample of a few TLDs from a batch.

ERD1: Based on AAPM TG‐40.^12^ Multiple TLDs can be cross‐calibrated simultaneously against an ion chamber measured dose at a reference depth in a solid phantom using a uniform radiation field.

IRD2: Can be established using classic H&D curve for one film for each new batch. Effects of batch film changes should be routinely assessed. Various techniques for obtaining a sensitometric and a dose–response curve are described in AAPM TG‐69^7^ for radiographic films and in AAPM TG‐55^8^ for radiochromic films.

WRD1: Testing to follow manufacturer recommendations.

ARD1: Based on AAPM TG‐69^7^ for radiographic films and on AAPM TG‐55^8^ for radiochromic films.

IRD3, IRD4, ARD2: Based loosely on AAPM TG‐40^12^ and clinical experience.

IRD5, IRD6: Based on AAPM TG‐40.^12^

ARD3: Based on AAPM TG‐40.^12^ Absolute dose calibration to be done if required.

IRD7, IRD8: Energy dependence of MOSFETs can be addressed by performing an absolute dose cross‐calibration in the beam energy and conditions they are intended to be used.^13^ Cross‐calibration for each beam quality against an ion chamber dose, as per AAPM TG‐51^3^ or TG‐43,^14^ following manufacturers’ recommendations.

ARD4: Absolute dose cross‐calibration in the beam energy and under conditions they are intended to be used.

IRD9: Linearity of the OSL detectors should be checked prior to use to assess the dose range at which the dosimeter remains linear.

IRD10, ARD5: Commercially available OSL detectors show minimal energy dependence in the megavoltage clinical energy range 6−25 MeV. Substantial energy dependence has been found in the kV range. Therefore, the same absolute calibration factor can be used in the megavoltage energy range, while an energy‐dependent calibration should be done for energies in the kV range.

IRD11: Linearity of the scintillating fibre dosimeter (SFD) should be checked prior to use to assess the dose range at which the dosimeter remains linear.

IRD12, ARD6: Commercially available SFDs show minimal energy dependence in the megavoltage clinical energy range 6−20 MeV. Substantial energy dependence has been found in the kV range. Therefore, the same absolute calibration factor can be used in the megavoltage energy range, while an energy‐dependent calibration should be done for energies in the kV range.

IRD13: The signal from plastic scintillators contains Cherenkov radiation generated in the light guide, which results in an undesired stem effect. A stem removal technique needs to be implemented to keep this effect below stated specifications.

**Table 3:** Basic measurement devices

Designator	Test	Performance	
		Tolerance	Action
Reference thermometer, barometer, hygrometer			
Initial use or following malfunction and repair			
IBM1	Calibration certificate	Characterize and document	
Biennial			
ABM1	Absolute calibration	Characterize and document	
Field thermometer, barometer, hygrometer			
Initial use or following malfunction and repair			
IBM2	Cross‐calibration	Characterize and document	
Biennial			
ABM2	Cross‐calibration	Characterize and document	
Spirit levels, self‐levelling laser system			
Initial use or following malfunction and repair			
IBM3	Calibration check	Characterize and document	
At each use			
EBM3	Calibration check	Characterize and document	

Note:

IBM1: Certificates are retained for reference devices.

ABM1: Calibration of reference devices to absolute values every 2 yr.

IBM2, ABM2: Field devices are compared (cross‐calibrated) against reference devices prior to initial use and every year except for barometers (6 months). Field devices are also checked against each other to identify damage. Frequency for barometers has changed from 3 months^12^ to 6 months based on local experience. Comparison of local barometer readings against the local airport system (corrected for altitude difference) is recommended. Digital barometers often require a correction factor that converts the digital readout into the true pressure. Barometers (analogue and digital) are checked every 6 months.^12^

IBM3, EBM3: Based on manufacturers’ recommendations. Certificates are retained for documentation. For a spirit level, its reading when placed on a flat or vertical surface should be the same when it is 180° rotated along an axis perpendicular to the surface. The verticality and horizontality of the lines projected by the self‐levelling laser should also be checked at each use.

**Table 4:** Automated beam scanning devices

Designator	Test	Performance	
		Tolerance	Action
Mechanical components			
Initial use or following malfunction and repair			
IBS1	Alignment	Characterize and document	
IBS2	Hysteresis	Characterize and document	
IBS3	Orthogonality/verticality	Characterize and document	
Annual			
ABS1	Positional accuracy	1 mm	2 mm
Detectors (ion chambers and diodes)			
Initial use or following malfunction and repair			
IBS4	Extracameral signal (stem effect)	0.5%	1.0%
IBS5	Linearity	0.5%	1.0%
IBS6	Leakage	0.5%	1.0%
Annual			
ABS2	Reproducibility of collection potential	0.5%	1.0%
Data acquisition/analysis			
Initial use or following malfunction, repair, or software upgrade			
IBS7	Scan speed insensitivity	Characterize and document	
IBS8	Scan mode (continuous versus step‐by‐step) insensitivity	Characterize and document	
IBS9	Agreement with static measurements	1.0%	2.0%
IBS10	Symmetry/flatness calculations	1.0%	2.0%
IBS11	Energy/Bremsstrahlung calculations	1.0%	2.0%
IBS12	Ionization‐to‐dose calculations	1.0%	2.0%

Note:

IBS1–IBS3: Based on clinical experience. Acceptance test criteria may be provided by the vendor as a guideline. A typical hysteresis check is to ensure that scanning in opposite directions leads to the same output.

ABS1: Based on clinical experience. Users may adapt and document criterion to local needs. Stated specifications from all current manufacturers are smaller than 0.5 mm.

IBS4: Based on IFS1.

IBS5: Based on similar criteria for IFS3.

IBS6: Based on IFS4 with looser criteria.

ABS2: Based on similar criteria for IFS5.

IBS7–IBS12: Tests based on clinical experience and may be modified to meet the user criteria. Tests may also be modified to follow the vendor's acceptance test criteria.

**Table 5:** Machine quality assurance devices

Designator	Test	Performance	
		Tolerance	Action
Diode and ionization chamber arrays			
Initial use or following malfunction and repair			
IMQ1	Positional accuracy, including distance to agreement (DTA) calculation	1.0 mm	2.0 mm
IMQ2	Signal reproducibility	Characterize and document	
IMQ3	Linearity (dose and dose‐rate)	Characterize and document	
IMQ4	Agreement with static measurements	1.0%	2.0%
IMQ5	Symmetry and flatness calculations	1.0%	2.0%
IMQ6	Energy dependence	Characterize and document	
Annual or biennial			
AMQ1	Relative array calibration	Characterize and document	

Note:

IMQ1–IMQ5: Based loosely on IBS5 to IBS11 and AAPM TG‐40.^12^ In addition, the manufacturers’ acceptance test procedures may be used to modify the user's criteria.

IMQ6: Based on clinical experience and manufacturer's recommendations. If devices are used across a range of beam energies, care must be taken to investigate their energy dependence and ensure that the appropriate calibration factors are applied for each measurement.

AMQ1: Based on clinical experience and manufacturer's recommendations. Array calibration ensures that all detectors in the array have the same sensitivity and thus eliminates response differences between individual detectors of the array. The resulting calibration factors may be energy‐dependent. Array calibration procedures and protocols are device‐specific and are provided by all vendors. Recalibration intervals depend on the type of detectors in the array (ion chamber or diode) and on the clinical workload. Vendor's guideline for array recalibration intervals can be followed.

**Table 6:** Treatment delivery quality assurance devices

Designator	Test	Performance	
		Tolerance	Action
Gantry mounting accessories			
Initial use or following malfunction and repair			
ITQ1	Gantry mount	Functional	
ITQ2	Alignment of detector central axis with crosshair	Characterize and document	
ITQ3	Detector plane position relative to the isocentre	Characterize and document	
Inclinometers			
Initial use or following malfunction and repair			
ITQ4	Inclinometer angle accuracy	0.5°	1.0°
Diode and ionization chamber arrays (2D and 3D)			
Initial use or following malfunction and repair			
ITQ5	Signal reproducibility	Characterize and document	
ITQ6	Linearity (dose and dose rate)	Characterize and document	
ITQ7	Agreement with static measurements (%/DTA)	1.0%/1 mm	2.0%/2 mm
ITQ8	Orientation of measured dose versus TPS dose map	Characterize and document	
ITQ9	Energy dependence	Characterize and document	
Annual or biennial depending on workload			
ATQ1	Agreement of device measurement with TPS	Analysis parameters: gamma index with 3% dose difference and 3 mm DTA Passing criteria: at least 95% of detectors with a *γ* ≤ 1	
ATQ2	Relative array calibration	Characterize and document	
ATQ3	Absolute cross‐calibration	1.0%	2.0%

Note:

ITQ1: Based on clinical experience and manufacturer's recommendations. It should be possible to attach the gantry mount accessory tightly on the gantry and to fix the detector array on it so that the detector does not move as the gantry and/or collimator rotate.

ITQ2, ITQ3: Based on clinical experience. With the detector array fixed on the gantry mount, the central axis of the detector array should align with the linac crosshair and the detector plane should be at isocentre. A 2 mm tolerance could be used here. Gross errors in the alignment and positioning can be corrected by adjusting the phantom setup in the TPS or by manipulation of device measurements. Also applies to relevant beam quality assurance devices.

ITQ4: Based on gantry/collimator angle indicators tolerance from AAPM TG‐40^12^ and AAPM TG‐142.^15^

ITQ5, ITQ6: Based on AAPM TG‐40.^12^ Manufacturers’ specifications can be used to set device‐specific tolerance and action levels.

ITQ7: Tolerances based on AAPM TG‐40^12^ and review of manufacturers’ specifications.

ITQ8: For each TPS, care must be taken to ensure that dose import parameters are setup correctly for TPS co‐ordinates to match those of the measuring device.

ITQ9: Same as IMQ6.

ATQ1: This is a consistency check based on clinical experience: a static field and an IMRT DQA plan can be created on the CT data set of the device in the TPS. These plans are periodically delivered on the device for consistency checks and analyzed with the gamma index parameters indicated. For the case of a static field, tighter tolerances can be used. However, the passing criteria can be adjusted locally based on the accuracy of the beam model of the TPS.

ATQ2: Same as AMQ1.

ATQ3: Based on clinical experience. Absolute dose cross‐calibration (at each beam quality) must be done following vendor's recommendations and against an ion chamber dose obtained following AAPM TG‐51,^3^ International Atomic Energy Agency (IAEA) TRS‐398,^6^ or AAPM TG‐148.^16^ After transfer of ion chamber dose to the device, the latter can be irradiated with the same beam used for calibration and the dose measured by the reference detector should agree with the ion chamber dose within indicated tolerance levels. This setup can also be used for routine checks of the absolute calibration of the device. Recalibration frequency is suggested by vendors and depends on workload for diode arrays. If devices are used across a range of beam energies, care must be taken to ensure that the correct calibration factors are applied.

**Table 7:** Phantom materials

Designator	Test	Performance	
		Tolerance	Action
Phantom materials			
Initial use			
IPM1	Electron density, homogeneity	Characterize and document	
IPM2	Dimensions of slabs or pieces	Characterize and document	

Note:

IPM1, IPM2: Inspection and radiographic verification prior to use is recommended. The tolerance depends on the intended use of the material and may be appropriately chosen by the user.

## CONFLICT OF INTEREST

The author declares no conflict of interest.
